# Factors associated with the unmet need for spacing and limiting family planning: Evidence from a national survey

**DOI:** 10.1371/journal.pgph.0007412

**Published:** 2026-10-09

**Authors:** Muhammad Khairul Alam, Rumana Rois

**Affiliations:** Department of Statistics and Data Science, Jahangirnagar University, Savar, Dhaka, Bangladesh; Western University of Health Sciences, CANADA

## Abstract

Traditional binary frameworks tend to combine the various aspects of unmet family planning needs into one general category. This study evaluates unmet family planning needs by first using conventional multinomial logistic regression to determine baseline demographic risk profiles. It then applies machine learning methods such as Random Forest and XGBoost to see if they can provide further insights into non-linear patterns and complex interactions between features that standard regression might miss. Using data from the Bangladesh Demographic and Health Survey (BDHS 2022), we built a multivariable multinomial logistic regression model. Additionally, we applied sophisticated ensemble classifiers such as Random Forest and XGBoost, paired with a synthetic hybrid resampling technique (SMOTE) to address severe class imbalance. Feature selection was conducted using the Boruta algorithm, and we used Shapley Additive Explanations (SHAP) to visualize complex global patterns and interaction effects. Bivariate analysis showed significant associations between unmet needs and maternal age, parity, wealth, residence, and decision-making power (p < 0.05). Multivariable logistic regression confirmed that having more children (4 or more) greatly increased the likelihood of unmet needs for spacing (aOR = 20.60) and limiting (aOR = 2.24). Wealth was notably protective against unmet needs for limiting, especially for the richest quintile (aOR = 0.48). Although SHAP analysis offered exploratory insights into complex, non-linear interactions between residence and factors such as parity and education, the predictive accuracy of tree-based models (Random Forest, XGBoost) was only moderate, with macro-averaged AUCs comparable to those of traditional regression. Therefore, population-level conclusions are most reliably drawn from the MLR model. The limitations of cross-sectional survey data as a diagnostic tool show that spacing needs are often fluid, transient, and shaped by relationships. Addressing the unmet need gap involves moving away from broad media campaigns toward localized counseling, ensuring stable rural spacing supplies, and involving husbands and mothers-in-law to turn reproductive healthcare into a shared household conversation.

## Background

Family planning (FP) is essential for reproductive health and significantly improves maternal health, child survival, and population dynamics. In Bangladesh, consistent investment in FP programs over many years has reduced fertility rates and caused demographic shifts, making the country an example of reproductive progress in a low-income setting. Key national efforts, such as community outreach, healthcare service delivery, and involving family planning workers, have played a crucial role in increasing contraceptive use and influencing reproductive decisions. However, a major public health issue remains: the unmet need for family planning. This term refers to women who want to delay or stop childbearing but are not using any contraceptive methods. Data from the Bangladesh Demographic and Health Survey (BDHS) indicate that while unmet need decreased in the 1990s, it has not been fully resolved. Further research shows that unmet need still leads to unintended pregnancies and unwanted fertility, especially in rural areas [1,2]. These results highlight that, despite high contraceptive prevalence, the unmet need for contraception continues to exist.

Socioeconomic inequality consistently impacts unmet family planning needs in Bangladesh. Women from poorer households face significantly higher unmet needs compared to those from wealthier families, mainly due to disparities in access to services, information, and resources. Urban–rural differences also persist, driven by variations in service availability, quality, transportation, and exposure to family planning messages [3]. These trends highlight ongoing structural and contextual barriers that hinder equitable access. Household and gender dynamics further influence contraceptive use and unmet needs. Research from Bangladesh and South Asia shows that women’s decision-making autonomy and spousal communication greatly correlate with contraceptive adoption [4,5]. When couples share reproductive decisions, unmet needs decline, emphasizing the importance of joint agency [6]. Conversely, extended family structures can restrict women’s reproductive choices, with evidence indicating that mothers-in-law often sway family planning decisions, reinforcing traditional fertility norms and discouraging contraception [4,7].

Knowledge, attitudes, and perceptions about contraception remain crucial factors influencing unmet needs. In Bangladesh, early national campaigns significantly increased awareness of family planning (FP) methods, as shown by historical data [8]. Still, misconceptions, fears of side effects, and inconsistent counseling persist, hindering effective contraceptive use. On the other hand, mass media exposure has a positive effect on contraceptive adoption, highlighting the importance of communication in influencing fertility behaviors [3]. Contact with family planning workers also strongly impacts fertility preferences and contraceptive choices, particularly in rural regions [1,9]. Unmet needs differ across various life stages and demographic groups. Adolescents and young women are at higher risk due to early marriage, limited autonomy, and barriers to youth-friendly FP services. A pooled DHS analysis from South Asia reports a high adolescent pregnancy rate often linked to unmet contraceptive needs [10,11]. In Bangladesh, marginalized and mobile populations face distinct challenges: Rohingya women in refugee camps have limited FP knowledge and hold negative attitudes toward contraception, affected by displacement and restricted access [12]. Meanwhile, women with migrant husbands experience occasional unmet needs due to spousal absence and irregular service access [13,14].

External shocks have intensified unmet needs in recent years. The COVID-19 pandemic disrupted family planning (FP) services in Bangladesh, limiting access to maternal and child health care and raising reproductive risks. Qualitative data reveal women’s experiences with service interruptions, mobility issues, and infection fears, especially among economically vulnerable groups. Long-term evaluations of FP programs in Bangladesh show that ongoing investment not only reduces fertility rates but also enhances women’s and children’s health and overall quality of life. These findings highlight the crucial developmental importance of effective family planning strategies.

While BDHS datasets are widely used to study family planning patterns, two key gaps remain. Firstly, many studies either combine ‘unmet need for spacing’ and ‘unmet need for limiting’ into a single binary category or analyze them separately, thereby masking the distinct socio-demographic and behavioral traits of each group. Traditional regression models are good at identifying linear, independent predictors but often miss the complex, overlapping factors influencing women’s reproductive health decisions. Conversely, advanced machine learning (ML) techniques can model complex nonlinear relationships and interactions among numerous features. This study’s main aim is not to declare a winner between traditional statistics and machine learning, but to evaluate whether algorithms such as Random Forest and XGBoost, with SHAP (Shapley Additive Explanations), offer additional insights beyond multinomial logistic regression risk profiles. Together, these methods aim to deepen understanding of the structural and socio-demographic factors affecting unmet family planning needs.

## Methods

### Ethics statement

This study conducts a secondary analysis of the Bangladesh Demographic and Health Survey (BDHS) 2022 dataset, which was obtained from the DHS Program website (https://dhsprogram.com) with permission. The BDHS data are publicly accessible and fully anonymized before release, with all identifying information removed. We accessed the dataset on [January 15, 2025]. Since this is a retrospective study utilizing secondary, de-identified data, additional ethical approval or informed consent was not required. Participants had previously provided informed consent during the original data collection carried out by the Bangladesh Bureau of Statistics in partnership with ICF under the DHS program. The Institutional Review Board (IRB) of ICF reviewed and approved the original BDHS 2022 survey protocol. At no point did the authors access individual identifiers during the analysis.

### Study design and data source

This study uses a quantitative cross-sectional design to explore factors associated with unmet family planning needs in Bangladesh. It analyzes data from the 2022 Bangladesh Demographic and Health Survey (BDHS), a nationally representative dataset gathered by the National Institute of Population Research and Training (NIPORT). The BDHS offers comprehensive details on household attributes, reproductive health, contraceptive practices, and sociodemographic factors.

### Study population and sampling

The BDHS 2022 dataset employs a stratified, two-stage sampling method encompassing both urban and rural regions of Bangladesh. The target population includes married women of reproductive age (15–49 years) who took part in the survey. After cleaning the data, only respondents with complete information on family planning and reproductive health indicators were included in the final analysis. The resulting sample size guarantees national representativeness and statistical validity.

### Accounting for survey design

To ensure the estimates are nationally representative and account for BDHS’s stratified, two-stage cluster sampling design, the analysis utilized complex survey weights. The individual sample weight (v005) was divided by 1,000,000 to normalize the probability weights. These normalized weights, along with the primary sampling units (clusters) and sample strata (v022/v023), were used to estimate multinomial logistic regression models, thereby adjusting for unequal selection probabilities and enabling the calculation of robust, unbiased standard errors.

### Outcome variable

This study’s outcome variable was unmet need for family planning, categorized into three distinct groups based on the revised DHS definition (variable v626a):

- No unmet need
- Unmet need for spacing
- Unmet need for limiting.

For the definition of the outcome variable, the analysis included married or in-union women of reproductive age. Women who were determined to be infecund or menopausal were excluded from the analytical sample, as they are not at risk for pregnancy and therefore do not contribute to the population-level unmet need for family planning [15].

### Covariates

Independent variables were chosen based on previous empirical studies and their availability in the BDHS 2022 dataset.

### Sociodemographic factors

These variables included the woman’s age, categorized into seven five-year groups (15–19 to –45–49), place of residence (urban or rural), education level of the woman and her partner (none, primary, secondary, higher secondary, or above), household wealth quintile (from poorest to richest), and occupation of both the woman and her partner (agriculture, professional/technical, sales/services, manual/domestic, and not working).

### Exposure to family planning information

Family planning information exposure was evaluated using BDHS variables v384a–v384h, which measure exposure to FP messages via mass media and interpersonal channels. A binary variable was created to indicate whether someone was exposed to at least one FP information source. Additional analyses examined the number of information sources to assess the robustness of the findings.

### Healthcare accessibility

Healthcare accessibility was evaluated using two DHS indicators: perceived distance to a health facility (v467d), which indicates whether distance was a significant barrier to access, and recent contact with family planning services, measured by receiving FP information from a health facility or provider (v384h). These indicators were combined into a composite variable categorizing access as poor, moderate, or good.

### Bivariate statistical analysis

To evaluate the initial relationships between the explanatory variables and the multi-class unmet-need categories, descriptive cross-tabulations were conducted. Pearson’s Chi-square tests of independence were calculated for each categorical factor to determine statistical significance, setting a threshold of p < 0.05.

### Addressing class imbalance

An examination of the target variable showed a significant class imbalance: most respondents fell into the “No Unmet Need” category (90.43%), while “Unmet Need for Spacing” (4.81%) and “Unmet Need for Limiting” (4.76%) were much smaller groups. To address the bias toward the majority class common in standard machine learning methods, a hybrid resampling technique combining synthetic and controlled sampling was used on the training data. This method integrated Synthetic Minority Over-sampling Technique (SMOTE), which creates artificial data points for minority groups, with down-sampling of the over-represented class. This process balanced the class distribution in the training set, enabling the algorithm to learn classification boundaries with equal emphasis.

### Feature selection using the Boruta algorithm

To filter out random noise and pinpoint the most robust predictors of unmet needs, the Boruta feature selection algorithm was applied. Boruta is a wrapper method centered around a Random Forest framework. It creates a randomized copy of all real features, termed “shadow features,” and compares the Z-scores of the actual predictors against the maximum Z-score among the shadow features. Only variables demonstrating structural importance higher than the maximum random shadow threshold were retained for final multi-class machine learning modeling.

### Machine learning frameworks and model training

Three distinct, structurally diverse algorithms were selected to model the balanced multi-class framework, running within a unified R modeling environment:

### Multinomial logistic regression (MLR)

A multinomial logistic regression served as the baseline model to assess the relationship between predictors and different unmet need categories compared to the reference category (no unmet need). It calculates category-specific log-odds as follows:


$$\begin{array}{c} \text{log}\left(\frac{P(Y=j)}{P(Y=\text{no unmet need})}\right)=\beta_{\text{0}}+\sum_{k}\beta_{k}X_{k}, \end{array}$$

where Y denotes unmet need category j, $X_{k}$are predictor variables, and $\beta_{k}$are regression coefficients. Results are presented as odds ratios with 95% confidence intervals. The goal of Multinomial Logistic Regression (MLR) is to create a baseline model that directly estimates the probability that a woman belongs to one of three outcome groups, using her socio-demographic traits.

**Random Forest (RF):** The goal of Random Forest (RF) is to build hundreds of independent decision pathways and average their outputs. This approach helps prevent the model from memorizing noise in the dataset and improves its ability to detect complex, non-linear patterns in women’s healthcare choices. Random forests are non-parametric ensemble methods that build a multitude of independent decision trees via bootstrap aggregation (bagging). It splits nodes using random feature subsets to lower variance and capture complex, non-linear interactions without strict distributional assumptions.

**XGBoost (Extreme Gradient Boosting):** Extreme Gradient Boosting (XGBoost) was applied for multi-class classification using a softmax objective. Essentially, the algorithm constructs decision trees one after another, with each new tree aimed at correcting the errors of the previous ones. It employs the ‘softmax’ function to generate probability scores for all three categories, ensuring each woman is classified into the most probable family planning status. An advanced, scalable gradient boosting framework that trains decision trees sequentially. Each successive tree is explicitly optimized to minimize the residual errors (loss function) of the preceding trees using gradient descent. To guarantee reproducibility, stability, and model generalization, internal validation was performed via repeated k-fold cross-validation with k = 5, 10, 15, and 20.

### Performance evaluation metrics

#### Model explainability and interpretability.

To enhance the interpretability of tree-based machine learning ensembles and avoid them functioning as opaque “black boxes,” SHapley Additive exPlanations (SHAP) were incorporated. Based on cooperative game theory, global SHAP importance features were derived by calculating the mean absolute SHAP values, which clearly measure each variable’s contribution to predictions. Additionally, localized SHAP dependence plots were created to analyze nonlinear interactions and structural relationships between geographic classification (urban/rural residence) and key predictors like parity, wealth, media exposure, and education. Because model discrimination for minority outcome classes remains limited, SHAP values are best interpreted as exploratory descriptions of the algorithm’s internal decision-making architecture rather than as established, population-level causal predictors.

#### Multivariable association modeling.

In addition to the machine learning extraction, a final parametric Multinomial Logistic Regression analysis was performed on the entire sample. The ‘No Unmet Need’ category served as the baseline reference. Adjusted Odds Ratios (aOR) and 95% Confidence Intervals (CI) were calculated to describe the risk profiles for the spacing and limiting unmet-need groups (p < 0.05).

### Performance evaluation metrics

Because of the substantial class imbalance, relying only on traditional accuracy may misrepresent a classifier’s true performance. Consequently, models were extensively evaluated on both unbalanced and balanced datasets, using a range of reliable diagnostic metrics to assess classification performance. In this context, classification quality reflects a model’s overall capacity to correctly distinguish the three target classes without favoring the majority class. The specific metrics used include:

- **Macro-Accuracy:** Assesses the average correctness of classifications computed independently for each class and then averaged, treating all classes equally regardless of their support size.
- **Balanced Accuracy:** Calculated explicitly as the arithmetic mean of the model’s overall Sensitivity and Specificity ($\frac{\text{Sensitivity}+\text{Specificity}}{\text{2}}$).
- **Sensitivity (Recall):** Measures how well the model identifies women with actual unmet family planning needs, indicating its effectiveness in capturing all missed cases in the population.
- **Specificity:** Evaluates performance in correctly classifying women with true met needs.
- **Precision:** Indicates the likelihood that women predicted to have an unmet need actually require services.
- **Macro and Micro F1-Scores:** The harmonic mean of precision and recall, capturing the model’s ability to optimize minority classes and providing a comprehensive measure of general classification performance, especially on imbalanced datasets.
- **Precision-Recall Area Under the Curve (PR-AUC) & Receiver Operating Characteristic (ROC) Area Under the Curve (AUC):** Assess the model’s ability to discriminate between classes across different thresholds for each outcome.

To prevent data leakage and ensure an unbiased evaluation, all synthetic resampling procedures (SMOTE-NC and down-sampling) were integrated into the preprocessing recipe and applied exclusively to the training data partitions. All validation folds and the final holdout test set maintained their original, real-world epidemiological distributions. All analyses were conducted in R using essential machine learning packages, and in STATA (version 17) for data wrangling.

## Results

### Distribution of unmet need for family planning

Women’s education was significantly associated with unmet need (χ² = 182.14, p < 0.001). The prevalence of unmet need for spacing varied across education levels, with women having secondary education reporting the highest prevalence (6.39%), followed by a slight decrease among those with higher education (5.98%). Limiting unmet need was highest among women with primary education (5.43%) and lowest among women with higher education (3.03%). Husband’s education also demonstrated a significant association (χ² = 117.32, p < 0.001). The unmet spacing need was highest among women whose husbands had secondary education (6.48%), whereas the limiting unmet need was lowest among women whose husbands had higher education (3.22%). Age showed a strong association with unmet need (χ² = 964.02, p < 0.001). Spacing unmet need was highest among adolescents aged 15–19 years (12.18%) and declined steadily with age. In contrast, unmet need remained elevated with age, peaking among women aged 30–34 years (7.41%) and remaining elevated among women aged 35–39 years (7.04%). Parity was significantly associated with unmet need (χ² = 260.55, p < 0.001). Unmet spacing need was highest among women with no children (8.67%), whereas limiting unmet need was highest among women with more than 3 children (6.52%). Women residing in rural areas had higher levels of both spacing (5.39%) and limiting (5.10%) unmet need compared with urban women (3.72% and 4.13%, respectively; χ² = 36.78, p < 0.001). The wealth index was significantly associated with unmet need (χ² = 30.54, p < 0.001). Unmet need for limiting and spacing was highest among women in the richest (5.68%) and poorest (5.56%) quintiles.

Decision-making for contraception use was significantly associated with unmet need (χ² = 69.60, p < 0.001). Spacing unmet need was highest among women reporting that “others” made contraceptive decisions (12.85%). Unmet need was highest among women who reported making their own decisions (6.75%). Women without media access had higher levels of unmet need for spacing (5.40%) and limiting (5.26%) compared with women with media access (4.39% and 4.42%, respectively; χ² = 18.22, p < 0.001). Health care access was modestly associated with unmet need (χ² = 12.91, p = 0.012). Women with good access had lower unmet need for spacing (2.83%) and limiting (3.46%) compared with those with poor access. Women reporting a distance barrier to health facilities had a higher limiting unmet need (5.23%) than those without a distance barrier (4.38%; χ² = 8.52, p = 0.014). Recent contact with family planning services was associated with unmet need (χ² = 6.75, p = 0.034), with slightly lower spacing unmet need among women with recent contact (3.20%) compared with those without (4.91%). Exposure to family planning information was modestly associated with unmet need (χ² = 11.60, p = 0.021). Limiting unmet need was lowest among women exposed to three or more information sources (3.54%). Women’s occupation was significantly associated with unmet need (χ² = 112.10, p < 0.001). Spacing unmet need was highest among non-working women (5.91%), whereas both spacing and limiting unmet need were comparatively lower among professional women (2.87% for each category). Husband’s occupation showed a strong association (χ² = 222.75, p < 0.001). Unmet need for spacing was highest among women whose husbands worked in sales/service occupations (6.62%), while limiting unmet need was highest among women whose husbands were not working (6.64%). Son’s preference was not significantly associated with unmet need (χ² = 1.60, p = 0.449). The distribution of spacing and limiting unmet need was similar across son-preference status. All of the above details are illustrated in Table 1.

**Table 1:** Distribution of unmet need for family planning according to respondents’ characteristics in Bangladesh, BDHS 2022 (N = 18,917).

Factors	Categories	No unmet need n (%)	Unmet need for spacing n (%)	Unmet need for limiting n (%)	Total (n)
Women’s education	No education	2277 (93.90)	32 (1.32)	116 (4.78)	**2425**
	Primary	4469 (91.62)	144 (2.95)	265 (5.43)	**4878**
	Secondary	7778 (88.66)	561 (6.39)	434 (4.95)	**8773**
	Higher	2585 (90.99)	170 (5.98)	86 (3.03)	**2841**
Husband’s education	No education	3713 (92.80)	94 (2.35)	194 (4.85)	**4001**
	Primary	4780 (90.21)	239 (4.51)	280 (5.28)	**5299**
	Secondary	5373 (88.37)	394 (6.48)	313 (5.15)	**6080**
	Higher	3243 (91.69)	180 (5.09)	114 (3.22)	**3537**
Son preference	No	15076 (90.47)	805 (4.83)	783 (4.70)	**16664**
	Yes	2033 (90.24)	102 (4.53)	118 (5.24)	**2253**
Parity	0-1 children	5563(89.78)	579 (9.34)	54 (0.87)	**6196**
	1–3 children	9002 (90.92)	312 (3.10)	671 (6.67)	**9985**
	4 or more children	2535 (92.65)	22 (0.80)	179 (6.54)	**2736**
Decision for contraception use	Self	1870 (88.75)	95 (4.51)	142 (6.74)	**2107**
	Husband	1515 (90.45)	100 (5.97)	60 (3.58)	**1675**
	Joint	13512 (90.76)	680 (4.57)	695 (4.67)	**14887**
	Others	212 (85.48)	32 (12.90)	4 (1.61)	**248**
Media exposure	No access	6990 (89.34)	423 (5.41)	411 (5.25)	**7824**
	Access	10119 (91.22)	484 (4.36)	490 (4.42)	**11093**
Women’s age (years)	15–19	1398 (87.48)	194 (12.14)	6 (0.38)	**1598**
	20–24	2763 (88.16)	318 (10.15)	53 (1.69)	**3134**
	25–29	3012 (88.02)	255 (7.45)	155 (4.53)	**3422**
	30–34	2944 (89.48)	102 (3.10)	244 (7.42)	**3290**
	35–39	2920 (91.97)	31 (0.98)	224 (7.06)	**3175**
	40–44	2200 (93.98)	5 (0.21)	136 (5.81)	**2341**
	45–49	1872 (95.66)	2 (0.10)	83 (4.24)	**1957**
Residence	Urban	6080 (92.16)	244 (3.70)	273 (4.14)	**6597**
	Rural	11029 (89.52)	663 (5.38)	628 (5.10)	**12320**
Wealth index	Poorest	3072 (91.87)	132 (3.95)	140 (4.19)	**3344**
	Poorer	3356 (90.24)	207 (5.57)	156 (4.19)	**3719**
	Middle	3427 (90.85)	155 (4.11)	190 (5.04)	**3772**
	Richer	3540 (90.15)	209 (5.32)	178 (4.53)	**3927**
	Richest	3714 (89.39)	204 (4.91)	237 (5.70)	**4155**
Woman’s occupation	Not working	10934 (89.10)	723 (5.89)	615 (5.01)	**12272**
	Agriculture	3561 (93.39)	82 (2.15)	170 (4.46)	**3813**
	Professional	394 (94.26)	12 (2.87)	12 (2.87)	**418**
	Sales/Service	838 (92.60)	34 (3.76)	33 (3.65)	**905**
	Manual/Domestic	1382 (91.58)	56 (3.71)	71 (4.71)	**1509**
Husband’s occupation	Not working	594 (89.59)	25 (3.77)	44 (6.64)	**663**
	Agriculture	4180 (94.85)	90 (2.04)	137 (3.11)	**4407**
	Professional	1931 (93.87)	66 (3.21)	60 (2.92)	**2057**
	Sales/Service	5921 (87.36)	449 (6.62)	408 (6.02)	**6778**
	Manual/Domestic	4483 (89.45)	277 (5.53)	252 (5.03)	**5012**
FP Information	No exposure	9996 (90.05)	550 (4.95)	555 (5.00)	**11101**
	1–2 sources	4937 (90.74)	242 (4.45)	262 (4.82)	**5441**
	3 + sources	2176 (91.62)	115 (4.84)	84 (3.54)	**2375**
Distance barrier	Not a barrier	9428 (90.97)	482 (4.65)	454 (4.38)	**10364**
	Barrier present	7681 (89.80)	425 (4.97)	447 (5.23)	**8553**
FP contact	No contact	16076 (90.35)	871 (4.90)	846 (4.75)	**17793**
	Recent contact	1033 (91.90)	36 (3.20)	55 (4.89)	**1124**
Health care access	Poor	7243 (89.82)	407 (5.05)	414 (5.13)	**8064**
	Moderate	9271 (90.73)	482 (4.72)	465 (4.55)	**10218**
	Good	595 (93.70)	18 (2.83)	22 (3.46)	**635**

In Fig 1, the Distribution of unmet need for contraception among currently married women (N = 18,954). The majority of women had no unmet need (90.43%), while 4.81% had an unmet need for spacing and 4.76% for limiting, indicating a substantial class imbalance in the outcome variable. Because the raw training dataset had severe class imbalance, with most observations in the No Unmet Need category and very few in the Spacing and Limiting category, a hybrid down-up resampling method was used. As shown in Fig 1, the original dataset was heavily skewed toward the majority class, potentially biasing standard machine learning models. The controlled hybrid resampling balanced the distribution, creating symmetrical target classes in the training set. This artificial balancing allowed machine learning algorithms to learn the feature boundaries of the minority unmet-need groups with equal importance.

**Fig 1:**
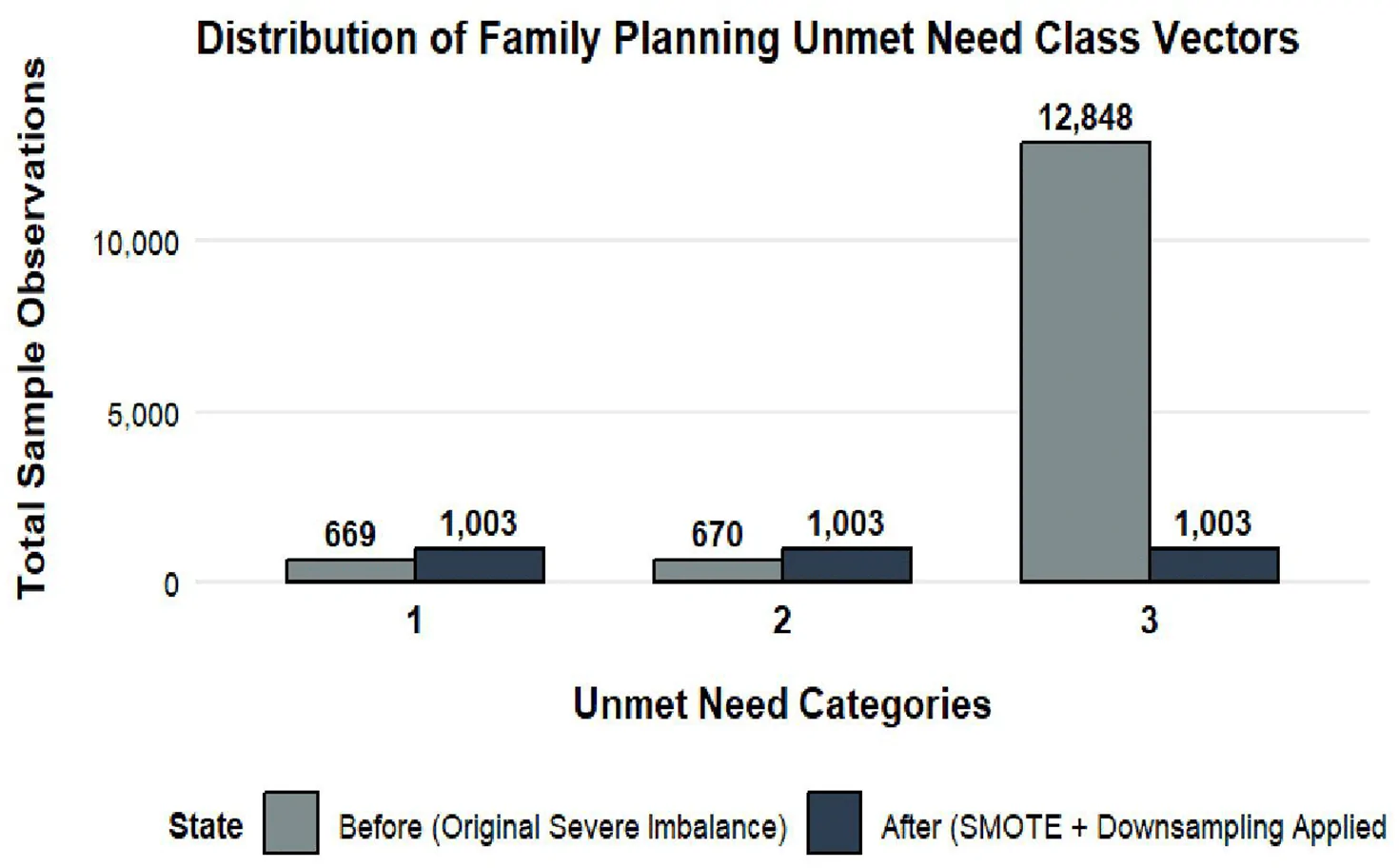
Distribution of Unmet Need for Contraception (before and after class imbalance).

### Boruta feature selection

Fig 2 shows the Boruta feature importance profile for factors linked to unmet family planning needs in Bangladesh. The analysis identified women’s age as the top predictor, with the highest median importance score among all variables. Other key factors included wealth index, residence location, women’s education, husband’s education, husband’s occupation, and media exposure, all of which were confirmed as significant by the Boruta algorithm. Predictors such as access to healthcare, distance barriers to health facilities, family planning information, and decision-making about contraceptive use played a moderately important role. These factors consistently received higher importance scores than shadow variables, underscoring their relevance in forecasting unmet family planning needs. Meanwhile, women’s occupation, family planning contact, and son preference showed lower importance scores but still exceeded the shadow-feature threshold, indicating they are relevant predictors. To identify truly important variables, we employed the Boruta algorithm. This technique generates ‘shadow variables’, randomized counterparts of our real data, serving as benchmarks. By shuffling the original variables, we produce a ‘null’ version that holds no actual information. When a real variable consistently exceeds the shadow variables in predicting the outcome, we can be sure its predictive power is genuine and not due to random variation.

**Fig 2:**
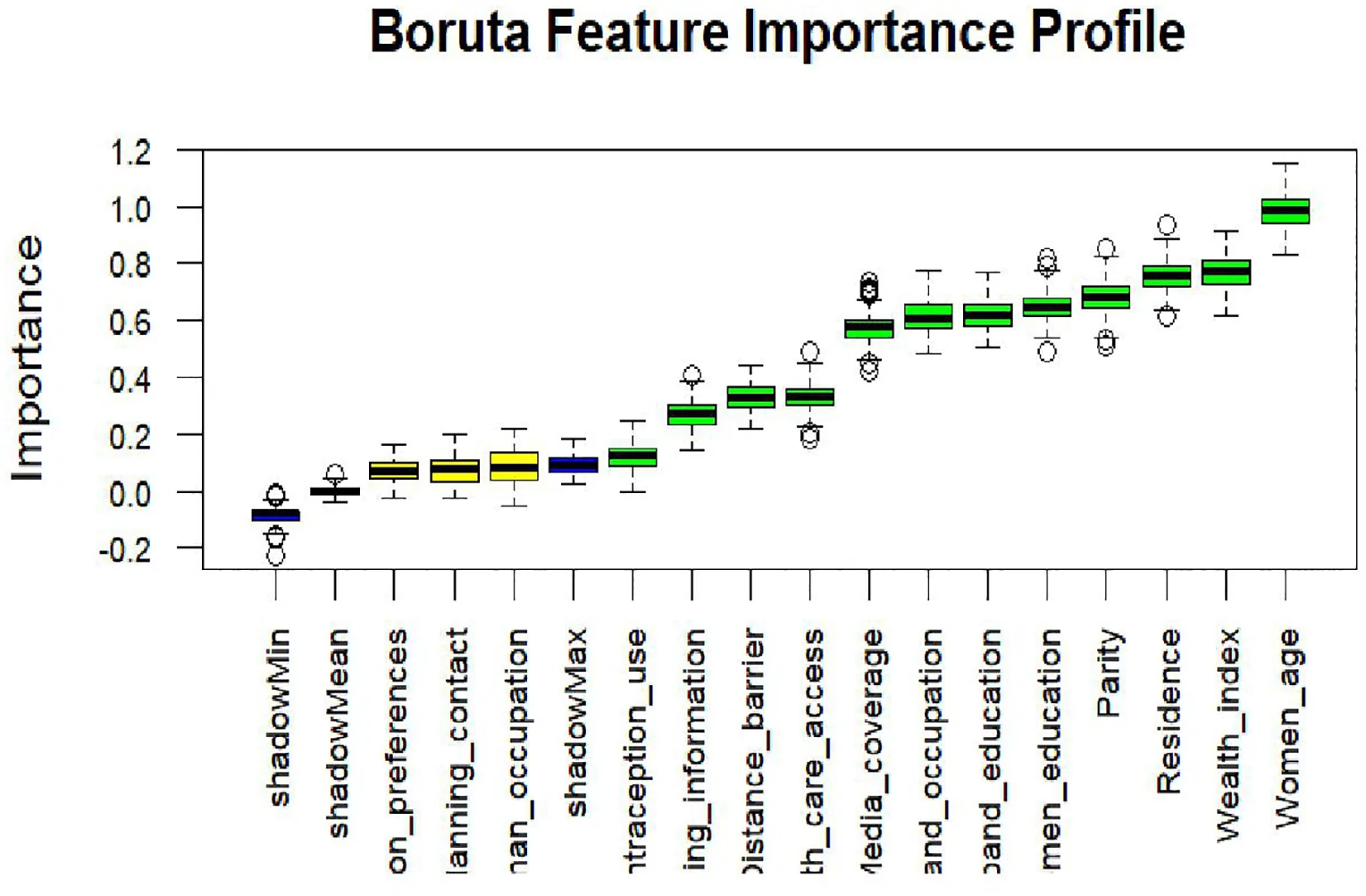
Boruta feature section for identifying factors associated with unmet need for family planning in Bangladesh.

### Machine learning model performance

The model’s predictive stability was evaluated through repeated cross-validation with different fold sizes [5,10,14,16], as shown in the supplementary performance comparison **(Table A in** S1 Appendix). Multinomial Logistic Regression consistently performed across all configurations, with an unbalanced macro-accuracy of around 0.905 and a balanced macro-accuracy between 0.397 and 0.404. Although Random Forest and XGBoost achieved higher balanced macro-accuracy of 0.511 and 0.507 in 20-fold cross-validation, all models displayed stable Precision-Recall AUC (PR-AUC) values, demonstrating that the resampling method effectively minimized the influence of extreme class imbalance on model convergence.

### Comprehensive evaluation of machine learning models

Table 2 presents the detailed performance metrics for multinomial logistic regression, Random Forest, and XGBoost models tested on both unbalanced and balanced datasets. Model performance was comprehensively evaluated across unbalanced and balanced datasets for Multinomial Logistic Regression (MLR), Random Forest, and XGBoost. On the original unbalanced data, all models exhibited poor predictive capacity for the minority classes, with balanced accuracies stagnating near 0.500 and baseline sensitivities of approximately 0.333. After applying our synthetic resampling strategy, model performance improved substantially. MLR achieved the highest balanced accuracy (0.645 ± 0.029) and sensitivity (0.560 ± 0.029) on the balanced data, marginally outperforming both Random Forest (balanced accuracy: 0.623; sensitivity: 0.519) and XGBoost (balanced accuracy: 0.624; sensitivity: 0.517). Specificity remained robust and consistent across all balanced models at approximately 0.73. Given its superior balanced accuracy and sensitivity, along with its inherent interpretability, MLR was retained as the primary model for evaluating the determinants of unmet need.

**Table 2:** Comprehensive performance evaluation of machine learning models on unbalanced and balanced datasets.

Model	Metric	Unbalanced Data (Mean ± SD)	Balanced Data (Mean ± SD)
Multinomial Logistic Regression	Balanced Accuracy	0.500 (±0.000)	0.645 (±0.029)
	Precision	0.860 (±0.143)	0.380 (±0.008)
	Sensitivity (Recall)	0.333 (±0.000)	0.560 (±0.029)
	Specificity	0.667 (±0.000)	0.729 (±0.018)
	F1-Score (Macro)	0.903 (±0.150)	0.291 (±0.018)
	F1-Score (Micro)	0.903 (±0.150)	0.291 (±0.018)
Random Forest	Balanced Accuracy	0.500 (±0.000)	0.623 (±0.034)
	Precision	0.906 (±0.007)	0.377 (±0.018)
	Sensitivity (Recall)	0.333 (±0.000)	0.519 (±0.056)
	Specificity	0.667 (±0.000)	0.728 (±0.028)
	F1-Score (Macro)	0.950 (±0.004)	0.327 (±0.026)
	F1-Score (Micro)	0.950 (±0.004)	0.327 (±0.026)
XGBoost	Balanced Accuracy	0.501 (±0.002)	0.624 (±0.018)
	Precision	0.595 (±0.251)	0.377 (±0.009)
	Sensitivity (Recall)	0.335 (±0.003)	0.517 (±0.029)
	Specificity	0.668 (±0.001)	0.731 (±0.010)
	F1-Score (Macro)	0.482 (±0.182)	0.331 (±0.018)
	F1-Score (Micro)	0.482 (±0.182)	0.331 (±0.018)

Fig 3 illustrates the overall exploratory SHAP feature importance ranking from the Random Forest model. It is important to note that these values reflect the model’s internal weighting strategy; within this framework, women’s age emerged as the most influential predictor. Its importance was approximately 5 times that of the second-most-significant predictor. Other key predictors included women’s occupation, parity, education, husband’s occupation, and residence, though their effects were considerably smaller than women’s age. Notably, categories related to women’s occupation and parity exhibited relatively high SHAP values, emphasizing that reproductive history and socioeconomic factors are vital in evaluating unmet family planning needs. Both women’s and their husbands’ educational levels also contributed significantly to the model outcomes, while residence-related factors had a moderate impact. Overall, the SHAP findings reinforce the Boruta feature selection results, identifying women’s age, parity, education, occupation, and residence as the main factors influencing unmet family planning needs in Bangladesh.

**Fig 3:**
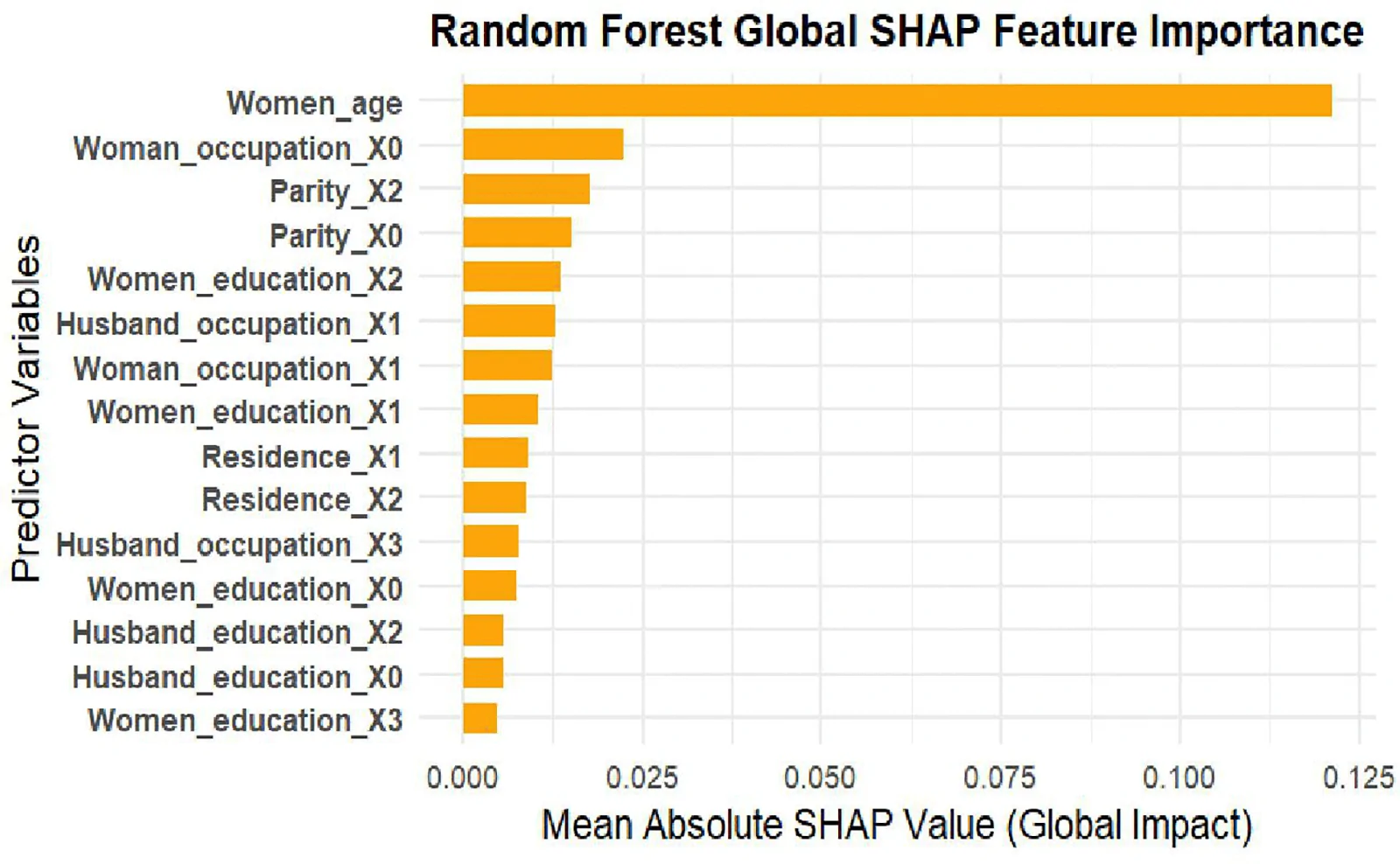
Global SHAP feature importance plot for the Random Forest model. Variables are ranked by their mean absolute SHAP values, which quantify each predictor’s average contribution to model predictions. Larger SHAP values indicate greater influence on the prediction of unmet family planning need. Variable Mapping: Woman_occupation_X0: Not working; Parity_X2: 4 and more children; Parity_X0: 0–1 Children; Women_education_X2: Secondary; Husband_occupation_X1: Agriculture; Woman_occupation_X1: Agriculture; Women_education_X1: Primary; Residence_X1: Urban; Residence_X2: Rural.

Fig 4 displays the exploratory interaction effects between residence and four key predictors. These SHAP dependence plots map how the model associates each predictor with unmet need across residential settings. In Panel A, there is a strong positive correlation between parity and SHAP values, indicating that women with more children are more likely to be predicted as having unmet family planning needs. The rising trend in SHAP values indicates that the likelihood of unmet need increases substantially for women with higher parity, regardless of whether they live in urban or rural areas. Panel B illustrates the interaction between wealth index and residence, with wealth showing a modest positive association with SHAP values, though this effect is smaller than that of parity. The notable overlap among residential groups indicates that wealth consistently affects unmet need across urban and rural populations. Panel C shows a positive association between women’s educational attainment and SHAP values, indicating that higher education is associated with greater influence on the model’s predictions. This suggests that education shapes unmet need patterns, and the differences observed between urban and rural women imply that the impact of education may vary by residential setting. Panel D illustrates the interaction between media exposure and residence. Although the overall effect size was small, women exposed to media generally had slightly higher SHAP values. The interaction pattern suggests that access to family planning information via the media may affect unmet need differently across residential areas.

**Fig 4:**
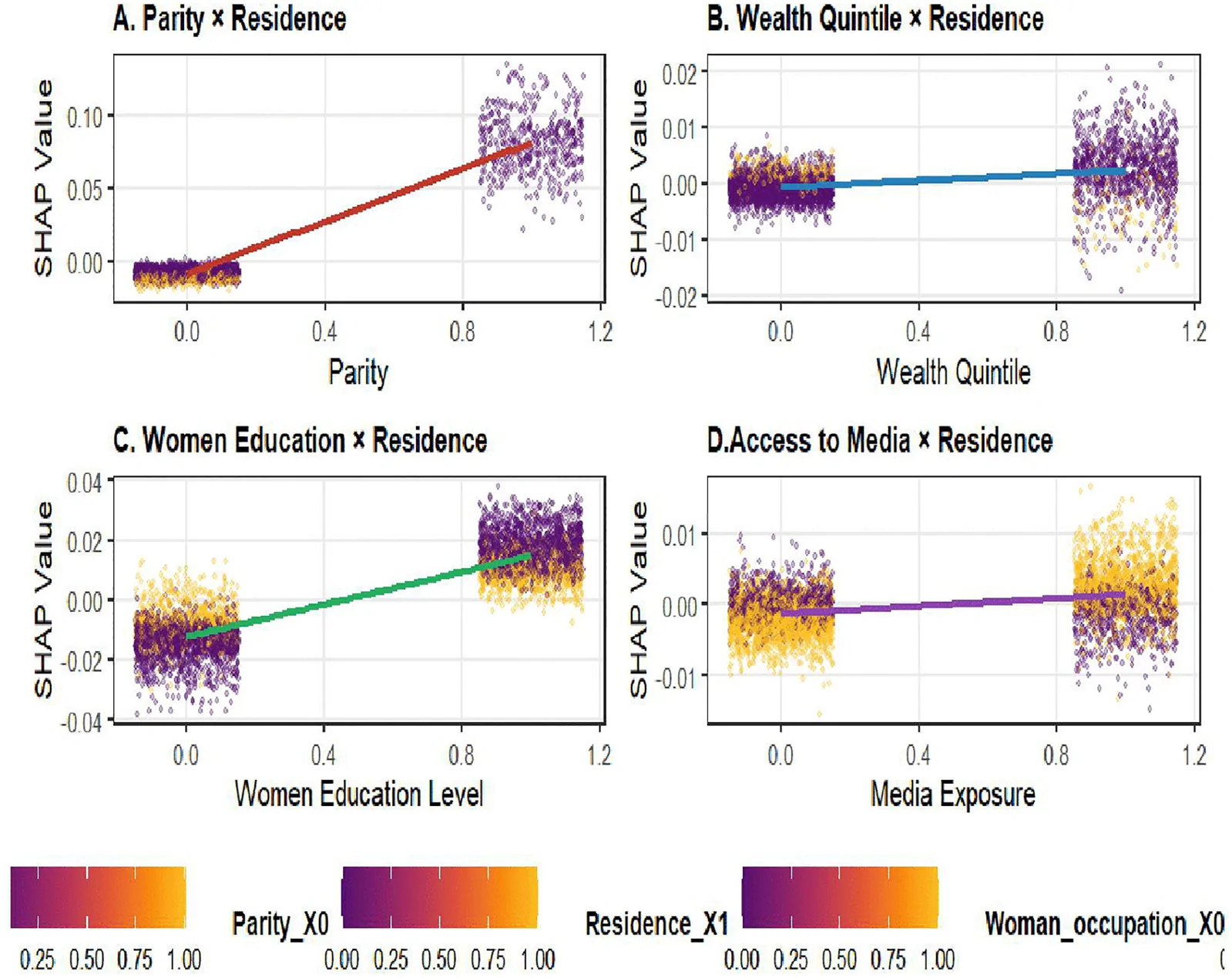
SHAP dependence plots showing interaction effects between residence and selected predictors in the Random Forest model. Panel A shows the interaction between parity and residence; Panel B illustrates wealth quintile and residence; Panel C depicts women’s education level and residence; and Panel D presents media exposure and residence. Positive SHAP values indicate increased contribution to the prediction of unmet need for family planning, while negative values indicate decreased contribution. Higher SHAP values represent a stronger influence on model predictions. Variable Mapping: Woman_occupation_X0: Not working; Residence_X1: Rural; Parity_X0: 0–1 Children.

The out-of-sample discriminatory performance of the Multinomial Logistic Regression, Random Forest, and XGBoost models across three family-planning unmet-need categories was evaluated using stratified 10-fold cross-validation. This analysis revealed highly consistent, overlapping ROC curves across all three models.

The model’s classification accuracy was assessed using macro-averaged ROC curves across the three unmet-need categories (Fig 5). The three models: MLR, Random Forest, and XGBoost showed comparable ability to distinguish between classes and consistently outperformed the chance diagonal. Among these, the unmet need for limiting displayed the weakest discrimination, with its ROC curve nearer the diagonal, while the unmet need for no unmet need exhibited the strongest, with a curve close to the top-left corner. This suggests that the models more readily identify features related to no unmet need than to limiting need. Although their overall performances were similar, the overlapping curves indicate that no single model clearly outperformed the others. Therefore, the MLR model was chosen for its interpretability and efficiency in elucidating sociodemographic factors.

**Fig 5:**
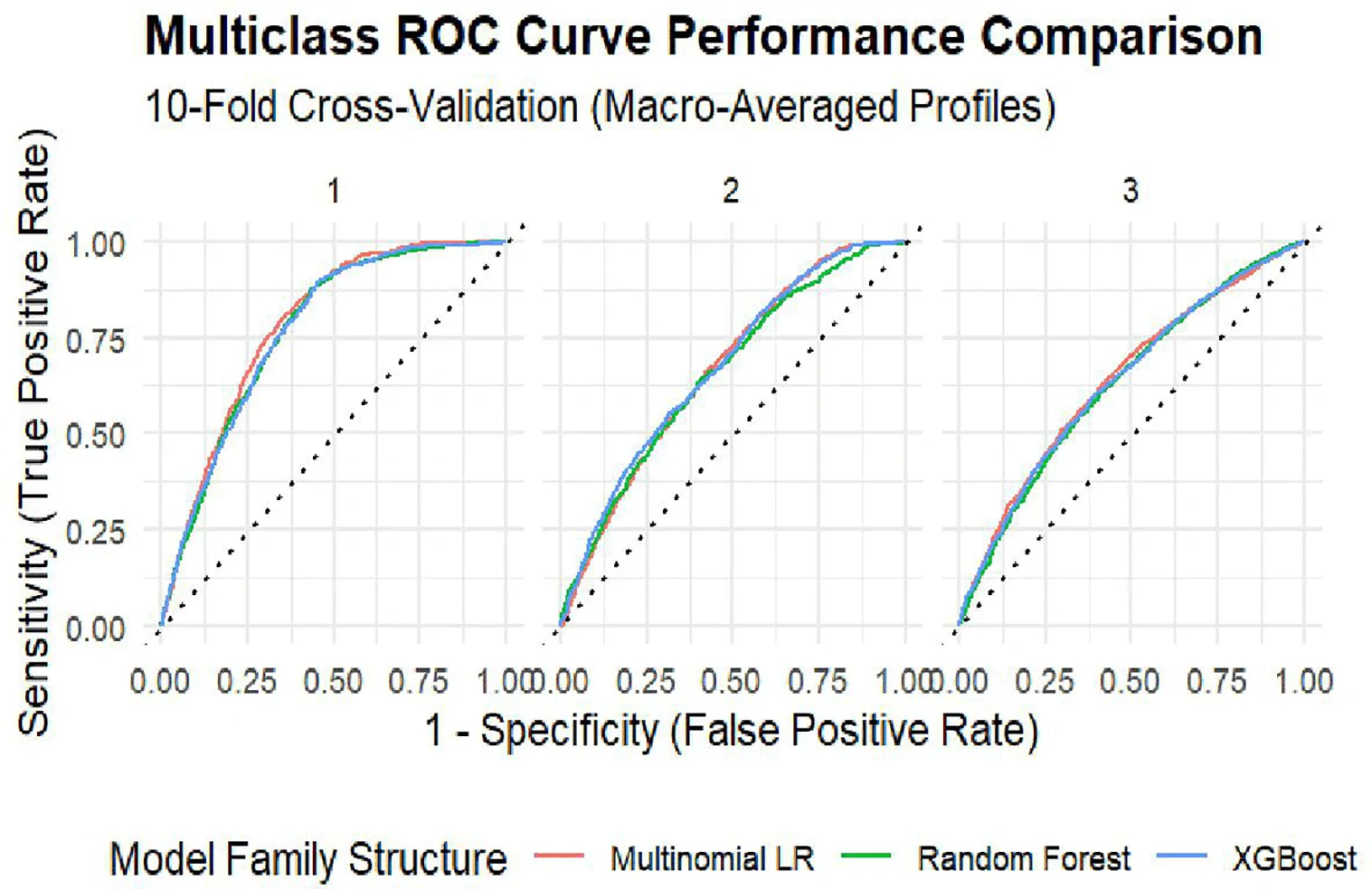
Multiclass ROC Curve Performance Comparison. The panels display the out-of-sample discriminatory capacity across the three target levels of family planning unmet need (Panels 1, 2, and 3), evaluated via a stratified 10-fold cross-validation design. The curves compare Multinomial Logistic Regression (red), Random Forest (green), and XGBoost (blue) against the diagonal baseline of random chance (dashed line). Notes: 1 “No unmet need”, 2 “Unmet need for spacing”, 3 “Unmet need for limiting”.

To further evaluate the reliability of the classification, calibration curves were generated for the Random Forest model (Fig A in S1 Appendix). These curves compare the predicted class probability vectors with the actual sample proportions across each unmet need category. The findings indicate that although the model generally captures overall trends in class distribution, it consistently deviates from the ideal diagonal calibration line, particularly in high-probability regions. This implies that while the model effectively identifies data patterns, its raw predicted probabilities should be interpreted with caution or recalibrated for clinical or programmatic use.

### Multivariable modeling

Table 3 shows the adjusted odds ratios (aOR) from the survey-weighted multinomial logistic regression, comparing the likelihood of unmet needs for spacing and limiting to the baseline of no unmet need. To enhance the stability of maximum-likelihood estimates, sparse parity categories were combined. Parity emerged as a significant, notably distinct predictor by unmet need type. Women with 2–3 children had significantly higher odds of unmet need for spacing (aOR = 9.57, 95% CI: 6.45–14.19) compared to those with 0–1 child. This difference was even more pronounced among women with 4 or more children, whose odds of unmet need for spacing were 20 times higher (aOR = 20.60, 95% CI: 10.19–41.46). In contrast, the risk of unmet need for limiting was significantly increased only among women with 4 or more children (aOR = 2.24, 95% CI: 1.25–4.02). Additionally, older maternal age was consistently associated with increased unmet need, with each additional year of age raising the odds for spacing (aOR = 1.66) and limiting (aOR = 1.63).

**Table 3:** Multinomial logistic regression analysis of factors associated with unmet need for spacing compared with no unmet need.

Factors	(Spacing vs. No unmet need) aOR (95% CI)	p-value	(Limiting vs. No unmet need) aOR (95% CI)	p-value
Women’s Education				
No Education (Ref)	**1.00**	**–**	**1.00**	**–**
Primary	**1.27 (0.76, 2.14)**	**0.364**	**1.15 (0.73, 1.81)**	**0.553**
Secondary	**1.07 (0.64, 1.78)**	**0.805**	**0.81 (0.52, 1.26)**	**0.355**
Higher	**0.91 (0.48, 1.73)**	**0.771**	**0.71 (0.43, 1.16)**	**0.174**
Husband’s Education				
No Education (Ref)	**1.00**	**–**	**1.00**	**–**
Primary	**0.95 (0.66, 1.37)**	**0.790**	**0.92 (0.68, 1.23)**	**0.567**
Secondary	**0.86 (0.60, 1.25)**	**0.431**	**0.83 (0.62, 1.10)**	**0.197**
Higher	**0.85 (0.52, 1.40)**	**0.530**	**1.24 (0.87, 1.77)**	**0.240**
Son Preference				
No Preference (Ref)	**1.00**	**–**	**1.00**	**–**
Prefers Sons	**0.93 (0.67, 1.30)**	**0.668**	**0.96 (0.75, 1.24)**	**0.773**
Parity				
0–1 Children (Ref)	**1.00**	**–**	**1.00**	**–**
2–3 Children	**9.57 (6.45, 14.19)**	**<0.001**	**1.08 (0.88, 1.33)**	**0.462**
4 + Children	**20.60 (10.19, 41.46)**	**<0.001**	**2.24 (1.25, 4.02)**	**0.007**
Decision for Contraception Use				
Decision: Self (Ref)	**1.00**	**–**	**1.00**	**–**
Decision: Husband	**0.85 (0.52, 1.39)**	**0.519**	**1.29 (0.89, 1.85)**	**0.174**
Decision: Joint	**0.89 (0.64, 1.24)**	**0.506**	**1.35 (1.04, 1.77)**	**0.026**
Decision: Others	**0.21 (0.06, 0.76)**	**0.018**	**0.70 (0.42, 1.17)**	**0.168**
Media Coverage				
No Access (Ref)	**1.00**	**–**	**1.00**	**–**
Has Access	**1.03 (0.82, 1.30)**	**0.805**	**1.34 (1.13, 1.58)**	**<0.001**
Woman’s Age				
Year	**1.66 (1.51, 1.82)**	**<0.001**	**1.63 (1.51, 1.76)**	**<0.001**
Residence				
Urban (Ref)	**1.00**	**–**	**1.00**	**–**
Rural	**0.93 (0.71, 1.23)**	**0.605**	**0.62 (0.50, 0.75)**	**<0.001**
Wealth Index				
Poorest (Ref)	**1.00**	**–**	**1.00**	**–**
Poorer	**0.76 (0.52, 1.11)**	**0.162**	**0.65 (0.49, 0.85)**	**0.002**
Middle	**1.11 (0.75, 1.64)**	**0.591**	**0.80 (0.60, 1.07)**	**0.139**
Richer	**0.92 (0.61, 1.37)**	**0.675**	**0.73 (0.54, 0.97)**	**0.033**
Richest	**1.06 (0.69, 1.64)**	**0.775**	**0.48 (0.35, 0.66)**	**<0.001**
Woman’s Occupation				
Not working (Ref)	**1.00**	**–**	**1.00**	**–**
Agriculture	**1.20 (0.87, 1.65)**	**0.275**	**1.48 (1.14, 1.92)**	**0.003**
Professional	**1.49 (0.51, 4.34)**	**0.461**	**1.55 (0.64, 3.75)**	**0.328**
Sales/Service	**0.72 (0.41, 1.27)**	**0.256**	**1.24 (0.85, 1.82)**	**0.260**
Manual/Domestic	**1.30 (0.82, 2.04)**	**0.263**	**1.53 (1.07, 2.18)**	**0.020**
Husband’s Occupation				
Not working (Ref)	**1.00**	**–**	**1.00**	**–**
Agriculture	**0.67 (0.33, 1.34)**	**0.256**	**1.48 (0.83, 2.62)**	**0.183**
Professional	**0.76 (0.35, 1.61)**	**0.467**	**1.21 (0.66, 2.20)**	**0.536**
Sales/Service	**0.79 (0.41, 1.51)**	**0.481**	**0.67 (0.40, 1.14)**	**0.143**
Manual/Domestic	**0.72 (0.37, 1.39)**	**0.327**	**0.75 (0.44, 1.28)**	**0.290**
Family Planning Information				
No exposure (Ref)	**1.00**	**–**	**1.00**	**–**
1-2 sources	**1.11 (0.86, 1.43)**	**0.404**	**1.06 (0.88, 1.28)**	**0.527**
3 + sources	**1.16 (0.79, 1.72)**	**0.444**	**1.16 (0.88, 1.52)**	**0.303**
Distance Barrier				
No Problem (Ref)	**1.00**	**–**	**1.00**	**–**
Big Problem	**0.87 (0.60, 1.26)**	**0.459**	**1.15 (0.86, 1.55)**	**0.336**
Family Planning Contact				
No Contact (Ref)	**1.00**	**–**	**1.00**	**–**
Contacted	**1.77 (1.03, 3.03)**	**0.037**	**0.95 (0.62, 1.45)**	**0.802**
Health Care Access				
Poor (Ref)	**1.00**	**–**	**1.00**	**–**
Moderate	**0.88 (0.61, 1.28)**	**0.513**	**1.30 (0.97, 1.74)**	**0.081**
Good	**0.36 (0.22, 0.60)**	**<0.001**	**1.79 (1.24, 2.60)**	**0.002**

Household wealth displayed a clear gradient, mainly protecting against unmet needs for limiting. Women in the richer (aOR = 0.73, 95% CI: 0.54–0.97) and richest (aOR = 0.48, 95% CI: 0.35–0.66) quintiles were significantly less likely to have unmet needs for limiting than those in the poorest quintile. However, wealth did not significantly predict unmet need for spacing. Women in rural areas had 38% lower odds of unmet need for limiting (aOR = 0.62, 95% CI: 0.50–0.75) than urban women. Interactions with healthcare access, media exposure, and decision-making gave mixed results. Contact with a family planning worker was associated with a higher odd of unmet spacing need (aOR = 1.77, 95% CI: 1.03–3.03), possibly indicating that workers identify existing needs. Media exposure was associated with a 34% increase in unmet need for limiting (aOR = 1.34, 95% CI: 1.13–1.58). Women with greater decision-making autonomy (others) had a 79% lower risk of unmet spacing need (aOR = 0.21, 95% CI: 0.06–0.76), highlighting the importance of intra-household decision dynamics.

## Discussion

This study used an explainable machine learning approach alongside traditional multivariable regression to explore the socio-demographic, economic, and behavioral groups with unmet family planning needs in Bangladesh. Moving beyond the conventional binary classification that merges spacing and limiting behaviors, our analysis identified specific programmatic gaps. Although the overall classification models achieved high macro-accuracy due to the prevalence of the majority class, detailed evaluation revealed very low multi-class AUC and recall scores for certain unmet needs, especially within the spacing group. This highlights a critical epidemiological insight: standard cross-sectional socio-demographic indicators typically found in large-scale surveys are insufficient to capture the complex, evolving, and relational nature of family planning decisions. A key insight from the SHAP global dependency trajectories is the complex, nonlinear, and multidirectional influence of economic status on family planning needs. Traditional linear models tend to oversimplify wealth as a straightforward protective factor against unmet needs [5]. In contrast, our localized analysis reveals a different picture. While the richest quintiles generally have lower rates of unmet needs, those in the wealthy group who do face gaps tend to show a strong positive trend, specifically for unmet needs related to limiting. Conversely, the poorest households exhibit highly positive SHAP values for unmet spacing needs.

A key discovery in this study is the notable divergence between the strong statistical support from multinomial logistic regression (MLR) and the exploratory patterns indicated by machine learning methods. For example, initial SHAP visualizations identified ‘son preference’ and ‘wealth’ as highly interactive and complex features influencing predictions. Yet, the survey-weighted MLR models, which properly account for complex survey design and provide sound statistical inference, showed a different picture at the population level. In detailed regression analysis, son preference was not statistically significant, while wealth consistently served as a protective factor, reducing the odds of women experiencing unmet needs for limiting. When SHAP and MLR findings conflict, priority should be given to the MLR estimates. The SHAP results for these variables should be viewed as artifacts of the algorithms’ internal, non-linear weighting, not as confirmed epidemiological effects. This mismatch underscores the risk of depending solely on black-box machine learning in public health policy, reaffirming that traditional survey-weighted regression remains the preferred method for valid population risk inference.

This socio-economic stratification highlights underlying structural inequities in resource distribution and access to methods between urban and rural areas [6]. Wealthier women tend to have greater health literacy and more financial resources to access terminal childbearing options or Long-Acting Reversible Contraceptives (LARCs). When their needs are unmet, these gaps are often due to system-level barriers such as local stockouts of contraceptives or healthcare disruptions caused by pandemics, which have disproportionately affected clinical infrastructure [11,17]. Conversely, women in poverty face persistent structural, geographic, and financial obstacles to obtaining basic, short-acting contraceptive methods like oral pills or injectables. A paradox emerges in media exposure patterns. Access to media shows positive SHAP values for the “No Unmet Need” group, confirming that mass communication is crucial for raising awareness about contraception [14,18]. However, it unexpectedly shows a negative trend in spacing needs and a strongly negative trend in limiting, indicating a significant disconnect in current health messaging. While broad media campaigns effectively highlight the basic benefits of family limitation once parity is reached, they are not designed to address the specific behavioral concerns of younger, lower-parity women. These younger women often have deep-rooted fears about contraceptive side effects and transient issues, bleeding anomalies, or perceived long-term subfertility associated with early-stage spacing methods [1].

Our analysis shows that women’s age significantly influences their reproductive trajectories, but this effect varies greatly across the life course [1,10]. The specific dependency patterns reveal that younger women have high, positive SHAP values for spacing, reflecting a strong desire to delay subsequent births early in marriage [4,19]. This tendency shifts as women age, with a dominant focus on limiting births once they reach a certain parity. When these age-related motives intersect with actual parity, key conflicts arise. Interpersonal dynamics within households are central to the success or failure of family planning in Bangladesh. A woman’s reproductive outcomes depend heavily on her husband’s occupation and education levels [8,14,20]. Effective spousal communication is known to be a key factor in encouraging couples to move from non-use to consistent contraceptive use [13,18]. In many rural areas, husbands control financial resources and serve as the main gatekeepers to healthcare access [14,21]. Additionally, a strong son preference remains a significant structural barrier, especially in limiting family size. While a preference for male children may not prevent early spacing, it strongly influences couples’ willingness to limit their family [16]. If a household has not yet reached its desired number of sons, the motivation to continue having children often outweighs the desire to stop. Regional studies support this, highlighting that mother-in-law figures often reinforce traditional pronatalist attitudes, further restricting young women’s autonomy within joint household settings [16,21,22]. The key methodological insight of this study is that tree-based machine learning models perform at a chance level when distinguishing among multiple outcomes, especially spacing needs. This indicates that traditional, static socio-demographic features from cross-sectional surveys like the BDHS do not provide sufficient information to accurately predict highly dynamic spacing behaviors. Since spacing needs are inherently fluid and context-dependent, qualitative research indicates that many women experience “intermittent needs” influenced by their husbands’ internal migration for work [23]. Furthermore, long-term data from Matlab’s maternal and child health programs show that intensive face-to-face home outreach is the most effective way to address unmet needs [7,9,24,25]. However, during disruptions such as the COVID-19 pandemic, these personalized outreach efforts often break down, leading to spikes in unmet needs that static variables cannot fully capture [11,17]. Similarly, specific settings such as the humanitarian crisis in Rohingya refugee camps present unique cultural and religious barriers that general national models overlook [12,26]. As a result, the SHAP dependency patterns in this study should be interpreted cautiously. Since SHAP explains the model’s internal logic rather than direct population dynamics, its interpretive power is limited by the model’s predictive capacity. Consequently, the non-linear trajectories and interactions identified should be viewed as exploratory patterns that help generate hypotheses for future targeted qualitative or longitudinal research, rather than definitive clinical predictors.

Our analysis identified key variables that can inform program design to reduce unmet family planning needs. The multinomial logistic regression revealed distinct demographic and socioeconomic factors affecting unmet need for spacing versus limiting. Notably, patterns linked to parity and age were evident that women with two or more children had significantly higher odds of unmet need for spacing, while unmet need for limiting was mainly observed among women with four or more children and those aged 40 or older. Socioeconomic and geographic factors also varied by unmet need type. Household wealth notably offered strong, graded protection against unmet need for limiting, but was not a significant predictor of spacing [25]. Rural residence was associated with a lower unmet need for limiting compared to urban areas. Finally, interactions with the healthcare system, particularly contact with family planning workers, were strongly associated with recognized unmet need for spacing, underscoring the complex factors influencing healthcare access and need recognition in these communities [3,27].

Our data details the demographic and socioeconomic profiles of women with unmet needs. To address these disparities, targeted interventions based on comprehensive family planning research are essential. When we place our findings in the context of broader evidence, several programmatic recommendations emerge. We observed that women in rural areas are less likely to have unmet needs for limiting; however, maintaining strong supply chains for spacing methods in these regions is vital, as disruptions can impede progress. Since general media exposure did not appear protective in our analysis, the literature supports localized, interpersonal counseling tailored to community-specific needs. Although our survey explored female decision-making autonomy, broader research underscores the importance of involving husbands and mothers-in-law to influence household fertility behaviors. To reduce parity-based disparities, programmatic data indicate the potential for developing digital health platforms and mobile interventions aimed at younger, low-parity women who may face stigma when accessing traditional clinics.

## Conclusions

In summary, our analysis shows that traditional multinomial logistic regression effectively estimates baseline risk probabilities and general sensitivity. However, tree-based machine learning models such as XGBoost and Random Forests offer additional insights by uncovering nonlinear patterns and complex socioeconomic influences on access to family planning. Moving forward, public health strategies should combine both approaches: using traditional regression to pinpoint vulnerable populations and applying machine learning to explore the detailed, multidimensional obstacles these groups encounter.

## Supporting information

S1 Appendix Fig A. Random Forest Multiclass Probability Calibration Curves. The panels illustrate the 10-fold cross-validated reliability profiles mapping predicted class probabilities against empirical sample proportions for each target category of family planning unmet need: (a) Target Class 1 (No unmet need), (b) Target Class 2 (Unmet need for spacing), and (c) Target Class 3 (Unmet need for limiting)**.** The dashed 45-degree diagonal line marks the ideal trajectory for a perfectly calibrated classifier, while the shaded region represents the 95% confidence interval. **Table A.** Performance measures include unbalanced and balanced macro-accuracy and unbalanced and balanced PR-AUC (mean ± SD) for Multinomial Logistic Regression, Random Forest, and XGBoost.
(DOCX)

## References

[1] Islam AZ, Mostofa MG, Islam MA. Factors affecting unmet need for contraception among currently married fecund young women in Bangladesh. Eur J Contracept Reprod Health Care. 2016;21(6):443–8. doi: 10.1080/13625187.2016.1234034 27676285

[2] Barkat-E-Khuda, Roy NC, Rahman DMdM. Unmet contraceptive need in Bangladesh: Evidence from the 1993/94 and 1996/97 demographic and health surveys. Asia Pac Popul J. 1999;14(2):1–10. doi: 10.18356/dca80c9f-en 12349402

[3] Callahan R, Becker S, Bloomberg H. Unmet need, intention to use contraceptives and unwanted pregnancy in rural bangladesh. Int Perspect Sex Reprod Health. 2014;40(1):4–10. doi: 10.1363/4000414 24733056

[4] Callahan R, Becker S. Unmet need, intention to use contraceptives and unwanted pregnancy in rural bangladesh. Int Perspect Sex Reprod Health. 2014;40(1):4–10. doi: 10.1363/4000414 24733056

[5] Bhatia B, Hossain S, Ghosh U, Salignac F. Reimagining gendered community interventions: The case of family planning programs in rural Bangladesh. Glob Health Res Policy. 2024;9(1):3. doi: 10.1186/s41256-023-00337-8 38225670 PMC10788984

[6] Shabuz ZR, Haque ME, Islam MdK, Bari W. Link between unmet need and economic status in Bangladesh: Gap in urban and rural areas. BMC Womens Health. 2022;22(1):176. doi: 10.1186/s12905-022-01752-8 35568941 PMC9107634

[7] Barham T, Champion B, Foster AD, Hamadani JD, Jochem WC, Kagy G, et al. Thirty-five years later: Long-term effects of the Matlab maternal and child health/family planning program on older women’s well-being. Proc Natl Acad Sci USA. 2021;118(28):e2101160118. doi: 10.1073/pnas.2101160118 34260397 PMC8409382

[8] Uddin J, Pulok MH, Sabah MN-U. Correlates of unmet need for contraception in Bangladesh: Does couples’ concordance in household decision making matter?. Contraception. 2016;94(1):18–26. doi: 10.1016/j.contraception.2016.02.026 26921638

[9] Joshi S, Schultz TP. Family planning and women’s and children’s health: long-term consequences of an outreach program in Matlab, Bangladesh. Demography. 2013;50(1):149–80. doi: 10.1007/s13524-012-0172-2 23212440

[10] Anik AI, Islam MR, Rahman MS. Association between socioeconomic factors and unmet need for modern contraception among the young married women: A comparative study across the low- and lower-middle-income countries of Asia and Sub-Saharan Africa. PLOS Glob Public Health. 2022;2(7):e0000731. doi: 10.1371/journal.pgph.0000731 36962420 PMC10021951

[11] Asadisarvestani K, Hulsbergen M. Experiences with family planning and abortion services during the Covid-19 pandemic: A qualitative study in Bangladesh, Iran and Netherlands. BMC Public Health. 2024;24(1):31. doi: 10.1186/s12889-023-17414-9 38166967 PMC10763037

[12] Abul Kalam Azad Md, Zakaria M, Nachrin T, Chandra Das M, Cheng F, Xu J. Family planning knowledge, attitude and practice among Rohingya women living in refugee camps in Bangladesh: A cross-sectional study. Reprod Health. 2022;19(1):105. doi: 10.1186/s12978-022-01410-0 35501903 PMC9063234

[13] Kamal SMM, Islam MdA. Interspousal communication on family planning and its effect on contraceptive adoption in Bangladesh. Asia Pac J Public Health. 2012;24(3):506–21. doi: 10.1177/1010539511399118 21659331

[14] Davies J, Mitra SN, Schellstede WP. Oral contraception in Bangladesh: Social marketing and the importance of husbands. Stud Fam Plann. 1987;18(3):157–68. doi: 10.2307/1966810 3617122

[15] Bradley SEK, Croft TN, Fishel JD. DHS analytical studies 25 revising unmet need for family planning [Internet]. 2012. Available from: www.measuredhs.com

[16] Pradhan MR, Mondal S. Examining the influence of mother-in-law on family planning use in South Asia: Insights from Bangladesh, India, Nepal, and Pakistan. BMC Womens Health. 2023;23(1):418. doi: 10.1186/s12905-023-02587-7 37553598 PMC10410985

[17] Roy N, Amin MB, Maliha MJ, Sarker B, Aktarujjaman M, Hossain E, et al. Prevalence and factors associated with family planning during COVID-19 pandemic in Bangladesh: A cross-sectional study. PLoS One. 2021;16(9):e0257634. doi: 10.1371/journal.pone.0257634 34547041 PMC8454962

[18] Sharan M, Valente TW. Spousal communication and family planning adoption: Effects of a radio drama serial in Nepal. Int Fam Plan Perspect. 2002;28(1):16. doi: 10.2307/3088271

[19] Poudel S, Dobbins T, Razee H, Akombi-Inyang B. Adolescent pregnancy in South Asia: A pooled analysis of demographic and health surveys. Int J Environ Res Public Health. 2023;20(12):6099. doi: 10.3390/ijerph20126099 37372686 PMC10298335

[20] Uddin J, Pulok MH, Sabah MNU. Correlates of unmet need for contraception in Bangladesh: Does couples’ concordance in household decision making matter?. Contraception. 2016;94(1):18–26. doi: 10.1016/j.contraception.2016.02.026 26921638

[21] Mumtaz Z, Salway S. Understanding gendered influences on women’s reproductive health in Pakistan: Moving beyond the autonomy paradigm. Soc Sci Med. 2009;68(7):1349–56. doi: 10.1016/j.socscimed.2009.01.025 19232810

[22] Acharya DR, Bell JS, Simkhada P, van Teijlingen ER, Regmi PR. Women’s autonomy in household decision-making: A demographic study in Nepal. Reprod Health. 2010;7:15. doi: 10.1186/1742-4755-7-15 20630107 PMC2914657

[23] Khan R, MacQuarrie KLD, Sultana M, Nahar Q. Intermittent needs for family planning among women with an internal migrant husband in Bangladesh: A qualitative study. Sex Reprod Health Matters. 2022;29(2):2097044. doi: 10.1080/26410397.2022.2097044 35939308 PMC9364705

[24] Rahman M, DaVanzo J, Razzaque A. Do better family planning services reduce abortion in Bangladesh?. Lancet. 2001;358(9287):1051–6. doi: 10.1016/S0140-6736(01)06182-7 11589936

[25] Phillips JF, Simmons R, Koenig MA, Chakraborty J. Determinants of reproductive change in a traditional society: Evidence from Matlab, Bangladesh. Stud Fam Plann. 1988;19(6 Pt 1):313–34. doi: 10.2307/1966627 3068840

[26] Abul Kalam Azad Md, Zakaria M, Nachrin T, Chandra Das M, Cheng F, Xu J. Family planning knowledge, attitude and practice among Rohingya women living in refugee camps in Bangladesh: A cross-sectional study. Reprod Health. 2022;19(1):105. doi: 10.1186/s12978-022-01410-0 35501903 PMC9063234

[27] Arends-Kuenning M, Hossain MB, Barkat-e-Khuda. The effects of family planning workers’ contact on fertility preferences: Evidence from Bangladesh. Stud Fam Plann. 1999;30(3):183–92. doi: 10.1111/j.1728-4465.1999.00183.x 10546310

