# Supplementary material for: Factors associated with the unmet need for spacing and limiting family planning: Evidence from a national survey
Source: PLOS Glob Public Health. 2026 Oct 9;6(10):e0007412. doi: 10.1371/journal.pgph.0007412 (PMC13652253; doi:10.1371/journal.pgph.0007412)
Supplement: Supplementary file 1 [file pgph.0007412.s001.docx]

**Supplementary materials**

**Table A. Performance comparison of machine learning models under repeated cross-validation**

| Folds | Model | Unbalanced Macro-Accuracy (Mean ± SD) | Balanced Macro-Accuracy (Mean ± SD) | Unbalanced PR-AUC (Mean ± SD) | Balanced PR-AUC (Mean ± SD) |
| --- | --- | --- | --- | --- | --- |
| 5 | Multinomial Logistic Regression | 0.906 ± 0.006 | 0.397 ± 0.009 | 0.388 ± 0.010 | 0.381 ± 0.008 |
| 5 | Random Forest | 0.906 ± 0.006 | 0.502 ± 0.015 | 0.381 ± 0.006 | 0.381 ± 0.009 |
| 5 | XGBoost | 0.905 ± 0.006 | 0.500 ± 0.009 | 0.389 ± 0.006 | 0.380 ± 0.009 |
| 10 | Multinomial Logistic Regression | 0.905 ± 0.007 | 0.402 ± 0.016 | 0.393 ± 0.016 | 0.384 ± 0.014 |
| 10 | Random Forest | 0.906 ± 0.007 | 0.507 ± 0.015 | 0.385 ± 0.013 | 0.388 ± 0.016 |
| 10 | XGBoost | 0.905 ± 0.007 | 0.502 ± 0.012 | 0.393 ± 0.014 | 0.386 ± 0.012 |
| 15 | Multinomial Logistic Regression | 0.906 ± 0.011 | 0.398 ± 0.012 | 0.391 ± 0.014 | 0.383 ± 0.016 |
| 15 | Random Forest | 0.906 ± 0.011 | 0.505 ± 0.017 | 0.384 ± 0.013 | 0.387 ± 0.011 |
| 15 | XGBoost | 0.905 ± 0.011 | 0.502 ± 0.010 | 0.395 ± 0.016 | 0.382 ± 0.012 |
| 20 | Multinomial Logistic Regression | 0.906 ± 0.011 | 0.404 ± 0.020 | 0.396 ± 0.020 | 0.389 ± 0.019 |
| 20 | Random Forest | 0.906 ± 0.011 | 0.511 ± 0.027 | 0.389 ± 0.018 | 0.394 ± 0.028 |
| 20 | XGBoost | 0.905 ± 0.011 | 0.507 ± 0.022 | 0.397 ± 0.017 | 0.386 ± 0.019 |

**Fig A. Random Forest Multiclass Probability Calibration Curves. The panels illustrate the 10-fold cross-validated reliability profiles mapping predicted class probabilities against empirical sample proportions for each target category of family planning unmet need: (a) Target Class 1 (No unmet need), (b) Target Class 2 (Unmet need for spacing), and (c) Target Class 3 (Unmet need for limiting). The dashed 45-degree diagonal line marks the ideal trajectory for a perfectly calibrated classifier, while the shaded region represents the 95% confidence interval.**
